# Identification and evaluation of potential microRNA markers for diagnostics in neurodegenerative diseases and correlation with other biochemical markers

**DOI:** 10.1371/journal.pone.0333801

**Published:** 2025-10-10

**Authors:** Richard Novobilský, Pavlína Kušnierová, Dominik Štěpán, Petra Bártová, David Stejskal, Michal Bar

**Affiliations:** 1 Clinic of Neurology, University Hospital Ostrava, Ostrava, Czech Republic; 2 Department of Clinical Neuroscience, University of Ostrava, Ostrava, Czech Republic; 3 Department of Clinical Biochemistry, Institute of Laboratory Medicine, University Hospital Ostrava, Ostrava, Czech Republic; 4 Institute of Laboratory Medicine, Faculty of Medicine, University of Ostrava, Ostrava, Czech Republic; University of Catania, ITALY

## Abstract

**Objectives:**

MicroRNAs (miRNA) are short, non-coding RNA molecules that play a crucial role in the development of organisms and are involved in various biological processes. They are considered potential biomarkers for many diseases, including neurodegenerative diseases. This study aimed to identify a set of microRNA targets that exhibited the greatest potential in successfully distinguishing and differentiating neurodegenerative diseases and to establish a correlation between selected miRNAs across different diagnostic groups.

**Methods:**

The study included the analysis of 126 patients. The patients were divided into five diagnostic groups – Alzheimer’s disease, non-Alzheimer’s dementia, Movement disorder, Dementia and movement disorder, and Healthy controls. The circulating RNA was isolated using the iCatcher Circulating cfRNA 1000 Kit with the iCatcher 12 automated isolator. The determination of microRNA was performed by TT-qPCR in the CFX96™ Real-Time Detection System. The concentrations of the remaining biomarkers were determined by ELISA. The statistical data were processed using MS Excel and MedCalc® software.

**Results:**

The following miRNAs were studied based on the primary screen for identification of potential microRNA targets and published literature data:hsa-miR-23a-3p, hsa-miR-29c-3p, hsa-miR-30b-5p, hsa-miR-142a-5p, hsa-miR-146a-5p, hsa-miR-151a-3p.A statistically significant correlation was identified between hsa-miR-29c-3p and hsa-miR-30b-5p, hsa-miR-30b-5p and hsa-miR-151a-3p, hsa-miR-23a-3p and hsa-miR-29c-3p, hsa-miR-23a-3p and hsa-miR-151a-3p, between hsa-miR23a-3p and hsa-miR-30b-5p, between hsa-miR-142a-5p and hsa-miR-146a-5p, hsa-miR-142a-5p and hsa-miR-151a-3p as well as between hsa-miR-146a-5p and hsa-miR-151a-3p.Significant differences were observed in hsa-miR-23a-3p and hsa-miR-29c-3p among different diagnostic groups. Compared to classical biomarkers of dementia, significant correlations were observed between plasmatic amyloid-β peptide 42 and hsa-miR-29c-3p, hsa-miR-142a-5p, hsa-miR-146a-5p, hsa-miR-151a-3p. Similar correlations were also found with the plasmatic amyloid-β peptide ratio of 42/40.

**Conclusions:**

The most promising microRNAs for differentiating among neurodegenerative diseases are hsa-miR-23a-3p and hsa-miR-29c-3p. Additionally, there is a correlation between hsa-miR-29c-3p and amyloid-β peptide and the ratio of amyloid-β peptide 42/40.While more robust studies are necessary, there could be a potential for utilizing this miRNA as a therapeutic agent in the future.

## Introduction

Neurodegenerative diseases represent a substantial and heterogeneous group of disorders, clinically manifesting as dementia syndromes, movement disorders, or a combination thereof. Currently, these disorders are approached from a biochemical perspective. They are caused by the pathological aggregation of specific proteins, which leads to apoptosis in certain groups of neurons. This process leads to atrophy and an irreversible loss of function [1].

Alzheimer’s disease (AD) is the most common cause of dementia in adults, accounting for 60% to 80% of cases [2]. The neuropathological hallmarks of the disease include the presence of β-amyloid (Aβ) plaques and the intraneuronal accumulation of neurofibrillary tangles formed by hyperphosphorylated tau protein [3]. The National Institute on Aging and the Alzheimer’s Association (NIA-AA) defines two sets of biomarkers: Core 1 and Core 2. Core 1 includes amyloid-beta (Aβ), which can be measured by cerebrospinal fluid (CSF) or positron emission tomography (PET), and plasma phosphorylated tau (p-tau), both of which reflect the early stages of AD. Core 2 includes total tau (t-tau), which tends to be positive in the later stages of the disease and may be a prognostic factor [4].

It is important to note that not all types of dementia are caused by the accumulation of Aβ; these are referred to as non-Alzheimer’s dementias. For instance, frontotemporal lobar degeneration (FTLD) is caused by the accumulation of proteins such as tau, TDP-43, and FUS [5]. A wide range of conditions that result in cerebral impairment have the potential to lead to dementia, including vascular dementia arising from strokes, dementia resulting from traumatic brain injuries, and dementia associated with neuroinflammation [6]. The clinical manifestation of these forms of dementia frequently deviates from that of AD, though in certain instances, distinguishing among the various forms of dementia can prove challenging.

Parkinson’s disease (PD) is a typical neurodegenerative movement disorder. The primary diagnostic method is a clinical examination, which identifies the typical symptoms of parkinsonism. According to the Movement Disorder Society (MDS), parkinsonism is characterized by bradykinesia in combination with either rest tremor, rigidity, or both [7]. Furthermore, dementia is prevalent among patients with PD, with estimations suggesting that up to 80% of patients may develop dementia as the disease progresses [8]. Neuropathologic hallmarks include the presence of α-synuclein aggregates in the central nervous system; however, the diagnostic lumbar puncture is not utilized in clinical practice [9].

There are several conditions that can cause parkinsonism which are not classified as Parkinson’s disease (PD) according to the Movement Disorder Society (MDS). These include multisystem atrophy, progressive supranuclear palsy, vascular parkinsonism, and post-inflammatory parkinsonism. Medications that are effective for PD are not beneficial for these conditions. Furthermore, dementia can occur in these cases, particularly in cases of Lewy Body Dementia, where it is a key diagnostic feature within the first year of clinical manifestations [10].

In addition to classical biochemical biomarkers, there is growing interest in the role of microRNAs in various pathological processes, including neurodegenerative disorders. MicroRNAs (miRNAs) are small, endogenous, non-coding single-stranded RNAs that regulate gene expression post-transcriptionally. They achieve this through interactions with the 3′ untranslated region (UTR) of target messenger RNAs (mRNAs) [11–13]. MiRNAs have been demonstrated to be implicated in a variety of biological processes and have been detected in multiple tissues, including cerebrospinal fluid, blood, urine, and saliva [11,14]. Furthermore, it has been demonstrated that these molecules exhibit a high degree of stability and resistance to extreme temperatures and pH fluctuations [15]. In addition to their role in apoptosis, neuroinflammation, and reactive oxygen species (ROS) production **–** factors that contribute to neurodegeneration **–** miRNAs have also been implicated in amyloidogenesis and tau pathology. Some miRNAs have been shown to regulate the degradation of the Amyloid Precursor Protein (APP), influence amyloid-beta (Aβ) metabolism, and enhance tau phosphorylation [16–19]. Evidence also exists for the role of miRNAs in regulating α-synuclein expression in Parkinson’s disease [20].

The primary objective of the present study was to identify a set of miRNA targets with the highest potential to successfully identify and differentiate neurodegenerative diseases. The secondary objective of this study was to establish a correlation between selected miRNAs and various biomarkers associated with neurodegeneration, as well as to investigate these relationships across different diagnostic groups.

## Materials and methods

### Patients

#### Identification of potential microRNA targets.

This retrospective study comprised 12 samples of CSF and 12 samples of serum obtained from the CSF Biobank of the University Hospital Ostrava between 2018 and 2020. The day of the data collection was October 1^st^, 2020. Authors had access to information that could identify individual participants.

The samples were subdivided into the following diagnosis groups: AD (n = 3), PD (n = 3) and controls (n = 6). The control group comprised samples of patients with normal cognitive performance, with no signs of parkinsonism and with normal CSF analysis.

In the AD group, all subjects had documented cognitive decline, with performance scores of less than 28 points on the Mini Mental State Exam (MMSE) at the time of lumbar puncture. The diagnosis was made using the NIA-AA research criteria for AD, with all subjects undergoing CSF analysis of triplets and magnetic resonance imaging (MRI) or computed tomography (CT) brain scans [21].

Conversely, the PD group exhibited the following characteristics: a clinical diagnosis of PD according to the MDS classification criteria, and no cognitive impairment (MMSE score of 28 or higher) [22].

The control group comprised patients who had undergone lumbar puncture for various reasons. These patients demonstrated no cognitive impairment (MMSE score of 28 or higher), and did not exhibit any movement disorders, focal lesions on brain MRI or CT scans, or inflammatory or autoimmune diseases, as determined by their CSF analysis.

A visual diagram of the selection process workflow is in S1 Fig.

#### Assay of selected miRNAs and other biomarkers.

For the purposes of prospective study, cerebrospinal fluid (CSF) and serum samples were collected over the course of three years, from May 1^st^, 2021 to December 1^st^, 2023, from patients attending University Hospital Ostrava, Czech Republic (n = 126, median age 69). The median age of the sample was 69.0 years (interquartile range [IQR] 59.0–74.0 years), and the proportion of female patients was 59.5%, with a median age of 69.0 years (IQR 59.0–76.5 years). The proportion of male patients was 40.5%, with a median age of 67.0 years (IQR 58.5–72.0 years). The following inclusion criteria were established for the study:

1. Participants are required to provide written informed consent to participate.
2. Brain imaging (utilising either CT or MRI) is to be conducted in order to exclude the presence of space-occupying brain lesions, including but not limited to tumours, brain contusions, multiple sclerosis, normal-pressure hydrocephalus, and significant post-ischemic or post-hemorrhagic lesions.
3. Furthermore, other potential causes of cognitive deficits, including but not limited to ion imbalance, anemia, vitamin B12 deficiency, Wilson’s disease, and thyroid disorders, must be ruled out through laboratory examinations.
4. Participants must have a Mini-Mental State Examination (MMSE) score of 25 out of 30 or lower, in addition to a documented duration of at least six months of clinical symptoms that affect daily activities.
5. A diagnosis of a neurodegenerative movement disorder is required, characterized by primary complaints other than dementia (such as Parkinson’s disease, multiple system atrophy, or progressive supranuclear palsy), accompanied by an MMSE score exceeding 25 out of 30 points.

The study excluded individuals under the age of 18. The control group was composed of patients who achieved a score higher than 28 out of 30 on the Mini-Mental State Examination (MMSE) and demonstrated no clinical signs of parkinsonism, such as tremor, rigidity, bradykinesia, hypokinesia, or gait disturbances, as evaluated by an experienced neurologist. A cut off score of 25 on the MMSE was established due to restrictions in reimbursement for AD treatment by Czech insurance. Additionally, one patient with severe dementia was unable to complete the MMSE due to non-cooperation.

The patients were categorized into five diagnostic groups based on their clinical and neuropsychological characteristics:

- Group 1: Alzheimer’s disease (AD); n = 38; median age 71.0 years, IQR = 62.0–78.0 years;
- Group 2: non-Alzheimer’s dementia; n = 33; median age 72.0 years, IQR = 65.0–78.0 years;
- Group 3: Parkinson’s disease (PD) and other movement disorder without cognitive deficit; n = 24; median age 62.5 years, IQR = 58.3–70.0;
- Group 4: combination of cognitive syndrome and movement disorders; n = 10; median age 71.0 years, IQR = 56.0–75.8 years;
- Group 5: healthy controls; n = 21; median age 56.0 years, IQR = 48.0–65.0 years.

Group 1 included patients with AD as defined by the National Institute on Aging and Alzheimer’s Association (NIA-AA) research criteria [21], even in the stage of mild cognitive impairment (MCI) without dementia (n = 6). 17 patients declined lumbar puncture, and their diagnosis was based on evaluation and monitoring by an experienced neurologist.

In Group 2, non-Alzheimer’s dementias included vascular dementia (n = 16), frontotemporal dementia (FTD; n = 8), Lyme neuroborreliosis (n = 3), alcohol-related dementia (n = 3), Creutzfeldt-Jakob disease (CJD; n = 2), primary progressive aphasia (n = 1).

Group 3 included patients with Parkinson’s disease diagnosed by an experienced neurologist, following the criteria established by the Movement Disorder Society (n = 14), multiple system atrophy (MSA; n = 4), progressive supranuclear palsy (PSP; n = 2), dystonia (n = 2), Huntington’s disease (n = 1) and essential tremor plus syndrome (n = 1).

Group 4 consisted of patients with Lewy body dementia (n = 6), MSA in the dementia stage (n = 2), PSP in the dementia stage (n = 1) and spinocerebellar ataxia (n = 1).

Group 5 included samples of patients who had undergone lumbar puncture for various reasons – depression and anxiety (n = 9), vertigo (n = 3), psychogenic movement disorder (n = 3), polyneuropathy (n = 3), cephalea (n = 2), transitory global amnesia (n = 1). All patients exhibited a normal cognitive profile (MMSE score of 28 or higher), had no movement disorders, no focal lesions on brain MRI or CT scans, and did not have any inflammatory or autoimmune diseases based on their CSF analysis.

A simple workflow diagram of the methodology is in S2 Fig.

### Samples

All samples of cerebrospinal fluid were obtained via lumbar puncture in the intervertebral space between L3/4, L4/5 and L5/S1 using an atraumatic needle and transferred into polypropylene tubes (Sarstedt, Nümbrecht, Germany). The standardised volume was 10 ± 1 mL.

Serum samples were collected in tubes containing a serum gel with clotting activator (Sarstedt), while plasma samples were collected in K_2_EDTA tubes (Sarstedt). CSF samples were subjected to a centrifugation process at 390 × g for a duration of 10 minutes at ambient temperature. In contrast, serum samples underwent a centrifugation procedure at 2500 × g for a duration of 6 minutes at a temperature of 4°C. Subsequent to this, both the CSF and serum/plasma samples were then aliquoted into at least three vials (0.3 ml per vial) and stored at 70°C until analysis.

### Analytical methods

#### Identification of potential microRNA targets.

RNA was isolated using the iCatcher Circulating cfRNA 1000 Kit (Cat. No. AC20100−36, CatchGene) using an automatic CatchGene isolator. The input volume for miRNA isolation from serum samples was 0.5 ml of serum and 0.5 ml of RNase free H_2_O, for miRNA isolation from CFS sample, 0.5 ml CSF and 0.5 ml RNase free H_2_O. The quality of RNA isolates was analysed spectrophotometrically using NanoDrop 2000 Spectrophotometer (ThermoFisher Scientific).

The isolated miRNA was subsequently analysed using the ID3EAL miRNA Knowledge Panel Biofluid (MIRXES) containing a total of 192 targets (including controls), of which 176 miRNA targets for screening. 5 μl of RNA isolate was used as the input volume for reverse transcription (RT).

#### Assay of selected miRNAs and other biomarkers.

The RNA isolation process was conducted utilising the iCatcher® Circulating cfRNA 1000 Kit, in accordance with the primary screening of microRNAs. Prior to the isolation procedure, it was essential to prepare the synthetic cell-miR-54-3p-TT (spike-in control) at a final concentration of 1x10⁹ miRNA copies/µl, which was subsequently employed as an isolation control. A total volume of 1 ml of serum was used for the isolation process, resulting in an elution volume of 30 µl.

Subsequently, the quantification of microRNAs was performed by means of a two-step quantitative polymerase chain reaction (TT-qPCR). This process integrates reverse transcription (RT) and quantitative polymerase chain reaction (qPCR) techniques. The reverse transcription reaction product, which is deoxyribonucleic acid (DNA), is then subjected to amplification by polymerase chain reaction (PCR) utilising two sequence-specific primers. A fluorescent SYBR label is utilised for detection. Monitoring of a melting curve during the reaction can be utilised for the detection of non-specific products.

The concentrations of additional biomarkers were determined in serum, plasma and cerebrospinal fluid were determined by ELISA methods using the following diagnostic kits: serum neurofilament light chain (NfL) by a high sensitivity ELISA assay (Nf-light serum ELISA, REF. 30210101, RUO, UmanDiagnostics AB); a cerebrospinal fluid NfL by diagnostic kit NF-light® ELISA CE (REF. 10–7001, UmanDiagnostics); total tau protein (t-tau) by Total-Tau-ELISA kit (REF. EQ 6531–9601-L, Euroimmun); phospho tau protein (p-tau) by pTau(181) ELISA kit (REF EQ-6591–9601-L, Euroimmun); Beta-Amyloid 1–42 in cerebrospinal fluid (CSF Aβ 1–42) by Beta-Amyloid (1–42) ELISA kit (REF. EQ 6521–9601-L, Euroimmun); Beta-Amyloid 1–40 in cerebrospinal fluid (CSF Aβ 1–40) by Beta-Amyloid (1–40) ELISA kit (REF. EQ 6511–9601-L, Euroimmun); Beta-Amyloid 1–42 in plasma (PL Aβ 1–42) by Plasma Beta-Amyloid (1–42) ELISA kit (REF. EQ 6521–9601, Euroimmun); Beta-Amyloid 1–40 in plasma (PL Aβ 1–40) by Plasma Beta-Amyloid (1–40) ELISA kit (REF. EQ 6511–9601, Euroimmun); Alpha-Synuclein (αS) by Alpha-Synuclein ELISA kit (REF EQ 6545–9601-L, Euroimmun) and Chitinase-3-like 1 (CHI3L1) by Quantikine ELISA Human Chitinase-3-like 1 Immunoassay (REF DC3L10, R&D Systems, USA&Canada). The detection limits were 0.4 ng/L for S NfL, 33 ng/L for CSF NfL, 28 ng/L for CSF tTau, 1.5 ng/L for CSF pTau, 41 ng/L for CSF Aβ 1–40, 6.5 ng/L for CSF Aβ 1–42, 5.9 ng/L for PL Aβ 1–40, 6.6 ng/L for PL Aβ 1–42, 19 ng/L for CSF αS and 3.55 ng/L for CHI3L1.

### Statistical methods

#### Identification of potential microRNA targets.

Basic processing of results and evaluation was performed using Microsoft Excel software. Measurement source data (Ct values) for each sample and miRNA target were processed according to the following procedure: only miRNA targets measured in the range of Ct 9–35 were evaluated as valid values; Ct values were normalized to interplate control (normalization to technical variability in measuring multiple PCR runs); values were normalized to RNA spike-in control (normalization to technical variability in multiple RT measurements); only miRNA targets that were detected in all samples of the group were included in the subsequent statistical analysis, other targets were excluded from the analysis; values were normalized to Global Mean (geometric mean of all measured values for a given sample, normalization of biological variability between samples). A total of 138 miRNA targets were analysed for the serum matrix after basic data processing. 38 targets were discarded due to low expression in the analysed samples. A total of 47 miRNA targets were analysed for the CSF matrix after basic data processing. 129 targets were discarded due to low expression in the analysed samples.

The statistical analysis was rendered using GenEx and Statistica software. Comparison of biological groups was performed using the non-parametric Mann-Whitney test. The values of differences in expression (fold-change) between individual groups were processed in Microsoft Excel, according to the formula: **Mean difference (*ΔΔ *C**_*t*_*) = Average (A) – Average (B)*, where *A* is biological group 1, *B* is biological group 2 and then: Fold – change = 2^-*ΔΔ Ct*^, if fold-change < 1, then: Fold – change = −1/2^-*ΔΔ Ct*^. The result of the group comparison is the expected Fold-change between the groups and the value of *p*, which indicates the degree of reliability of the result.

MiRNA targets with a fold-change > 1.5x (i.e., at least 50% difference between groups) and a p-value < 0.05 (Bonferroni correction for multiple determinations neglected) were marked as significant. The GeNorm and Normfinder tools were used to select the endogenous control from the measured data, from which the individual results were compared and the 5 best potential endogenous controls were selected for each matrix.

#### Assay of selected miRNAs and other biomarkers.

A power analysis was conducted prior to the commencement of the study. The analysis employed an anticipated effect size of f = 0.4, a significance level of α = 0.05, and a power of 0.80. It was determined that, in accordance with these parameters, the total sample size would be 80 subjects, with 16 participants per group.

The statistical analysis was performed using Microsoft Excel software. miRNA targets measured in the range of Ct 9–35 were evaluated as valid values. Ct values of samples were normalized to interplate control (IPC) and the spike-in control. Technical normalization of Ct values was performed according to the formula: Ct_sample_ = Ct_Sample median_ – Geomectric mean IPC of the plate* + Geomectric mean IPC of all plates (*same as normalized sample). Biological normalization of Ct values was performed using an exogenous “spike-in” control according to the formula: ∆Ct_sample_ = Norm Ct miR† - Norm Ct biocontrol† (i.e., † same sample). For further statistical treatment of the data, relative values obtained according to the formula: RQ = 2 ^-∆C^. Fold-Change between the 2 groups between the 2 groups was calculated similarly as above.

The other statistical analysis was performed in MedCalc statistical software (version 22.021) and the significance level was set to 0.05. Numerical variables are presented as medians and interquartile ranges (IQR), categorical variables are presented as absolute and relative frequencies (%). The significance of between-group differences is tested with Mann-Whitney test or Kruskal-Wallis test. Assessment of relationships of numerical variables is performed with the Spearman’s correlation coefficient and its test of significance.

The diagnostic utility of specific biomarkers was assessed using receiver operating characteristic (ROC) curve analysis. For each biomarker, sensitivity and specificity (along with their 95% confidence intervals [CIs]) were calculated, comparing the control group to the group affected by neurodegenerative processes. A biomarker with an area under the curve (AUC) greater than 0.8 was deemed to possess adequate diagnostic power, while a biomarker with an AUC less than 0.5 was considered to lack diagnostic power. The significance level for statistical tests was set at p = 0.05.

### Ethics approval

Written informed consent was obtained from all participants included in the study. The study was approved by the University Hospital Ostrava Ethics Committee as part of two projects: the first, entitled ‘CSF biobanking’ (reference number 22/2018), and the second, entitled ‘Laboratory biomarkers of neurodegenerative diseases’ (reference number 340/2021).

## Results

### Identification of potential microRNA targets

When comparing individual diagnostic groups, hsa-miR-497-5p, hsa-miR-328-3p, and hsa-miR-4732-3p were statistically significantly upregulated in serum in AD vs. controls; hsa-miR-4732-3p, hsa-miR-222-3p, hsa-miR-133a-3p, hsa-miR-625-5p, and hsa-miR-199a-5p in PD vs. control, and hsa-miR-4732-3p, hsa-miR-496-5p, hsa-miR-122-5p, hsa-miR-616-3p, hsa-miR-199a-5p, hsa-miR-19a-3p, hsa-miR-139-3p, hsa-miR-222-3p, hsa-miR-625-5p, and hsa-miR-99a-5p in AD + PD vs. control (see S1 File, Table 2).

**Table 2:** Correlations between microRNAs (miRNAs) and other biomarkers of neurodegeneration.

		hsa-miR-23a-3p	hsa-miR-29c-3p	hsa-miR-30b-5p	hsa-miR-142a-5p	hsa-miR-146a-5p	hsa-miR-151a-3p
CSF t-tau (ng/L)	r_S_ P n	−0.213 0.099 61	−0.158 0.225 61	−0.307 0.014 63	−0.139 0.276 63	−0.022 0.866 61	−0.207 0.103 63
CSF p-tau (ng/L)	r_S_ P n	−0.132 0.319 59	−0.076 0.566 59	−0.076 0.563 61	−0.222 0.085 61	−0.086 0.516 59	−0.167 0.198 61
CSF Aβ 1–42 (ng/L)	r_S_ P n	0.045 0.729 61	0.047 0.719 61	−0.039 0.764 63	0.010 0.936 63	−0.081 0.536 61	−0.033 0.799 63
CSF Aβ 1–40 (ng/L)	r_S_ P n	−0.231 0.127 45	−0.180 0.243 44	−0.287 0.053 46	−0.187 0.213 46	−0.228 0.137 44	−0.276 0.063 46
CSF Aβ 1–42/Aβ 1–40 cut off 0.095	r_S_ P n	−0.055 0.722 45	0.117 0.451 44	−0.064 0.673 46	0.017 0.910 46	−0.079 0.609 44	−0.058 0.703 46
PL Aβ 1–42 (ng/L)	r_S_ P n	−0.056 0.689 53	−0.555 <0.001 52	−0.221 0.109 54	0.612 <0.001 54	0.548 <0.001 52	0.448 <0.001 54
PL Aβ 1–40 (ng/L)	r_S_ P n	0.223 0.141 45	0.243 0.111 44	−0.008 0.960 46	−0.001 0.995 46	−0.120 0.438 44	0.102 0.502 46
PL Aβ 1–42/Aβ 1–40	r_S_ P n	−0.174 0.254 45	−0.720 <0.001 44	−0.119 0.432 46	0.522 <0.001 46	0.551 <0.001 44	0.365 0.013 46
CSF αS (ng/L)	r_S_ P n	−0.249 0.099 45	−0.238 0.120 44	−0.363 0.013 46	−0.099 0.511 46	−0.061 0.692 44	−0.178 0.237 46
CSF NfL (ng/L)	r_S_ P n	−0.006 0.961 64	0.143 0.260 64	−0.012 0.925 66	−0.193 0.121 66	−0.102 0.428 63	−0.137 0.274 66
Serum NfL (ng/L)	r_S_ P n	0.041 0.735 72	0.145 0.225 72	−0.061 0.607 74	−0.069 0.557 74	−0.137 0.252 72	−0.076 0.521 74
Serum CHI3L1 (ng/L)	r_S_ P n	−0.116 0.373 61	0.004 0.979 61	−0.211 0.098 63	0.088 0.492 63	0.007 0.960 60	−0.070 0.585 63

*Note: The values represent the Spearman’s correlation coefficient (r*_*S*_ *), the p-value of its test of significance (P) and the number of patients (n).*

At the same time, when the AD and PD groups were compared, 18 up-regulated and 12 down-regulated serum miRNA targets were selected for further study (see S1 File, Table 3).

**Table 3:** Comparison of diagnostic groups in serum hsa-miR-23a-3p and hsa-miR-29c-3p.

	hsa-miR-23a-3p		hsa-miR-29c-3p	
	n	Median (Min; Max)	n	Median (Min; Max)
Diagnostic group				
1	23	0.0063 (0.0025; 0.0733)	24	0.0036 (0.0011; 0.1280)
2	22	0.0142 (0.0028; 0.0862)	22	0.0063 (0.0016; 0.1670)
3	17	0.0102 (0.0028; 0.0616)	18	0.0110 (0.0017; 0.1230)
4	8	0.0276 (0.0034; 0.0777)	8	0.0696 (0.0020; 0.2280)
5	15	0.0158 (0.0063; 0.0516)	13	0.0031 (0.0014; 0.0067)
*p*-value of Kruskal-Wallis test	0.049		0.043	
Post-hoc analysis (Conover)	(1-4-5), (4−1), (5−1)		(1−4), (4-1-5), (5−4)	

*Note: Group 1: Alzheimer’s Disease; 2: non-Alzheimer’s dementia; 3: Parkinson’s Disease; 4: combination of dementia and movement disorder; 5: healthy controls*.

A similar miRNA screening, this time in the cerebrospinal fluid matrix, yielded fewer suitable miRNAs for further research, none of which were statistically significant.

### The selection of the appropriate miRNAs

Based on identification of potential miRNA targets and the published literature data, the study focused on the following miRNAs: hsa-miR-23a-3p, hsa-miR-142a-5p, miR-151a-3p, hsa-miR-146a-5p, hsa-miR-29c-3p, hsa-miR-30b-5p. These were selected because they consistently appeared dysregulated in neurodegeneration across independent datasets.

A dysregulation of hsa-miR-23a-3p [23,24] and hsa-miR-142a-5p [25] were found to be in patients with AD, while hsa-miR-151a-3p was found to be dysregulated in patients with both PD and AD [23,26]. Furthermore, hsa-miR-146a-5p was found to be dysregulated in patients with mild cognitive impairment (MCI) and AD and PD [27,28]. Consequently, aberrant expression in AD, MCI and PD has been described for hsa-miR-29c-3p [29–32] and aberrant expression in AD and PD for hsa-miR-30b-5p [33,34]. cel-miR-54-3p was used as an exogenous control. The results of the identification of potential miRNA targets are presented in S1 File.

### Analysis of dependence of selected miRNA and other biomarkers

An analysis was conducted to ascertain the dependence of selected microRNAs and other biochemical markers found in serum, plasma and cerebrospinal fluid. Significant correlations were identified between hsa-miR-23a-3p and hsa-miR-29c-3p, hsa-miR-30b-5p, hsa-miR-142a-5p, hsa-miR-146a-5p, and hsa-miR-151a. A statistically significant correlation was identified between hsa-miR-29c-3p and hsa-miR-30b-5p (P < 0.001), hsa-miR-30b-5p and hsa-miR-151a-3p (P < 0.001), hsa-miR-23a-3p and hsa-miR-29c-3p (P < 0.001), hsa-miR-23a-3p and hsa-miR-151a-3p (P < 0.001), between hsa-miR23a-3p and hsa-miR-30b-5p (P < 0.001), between hsa-miR-142a-5p and hsa-miR-146a-5p (P < 0.001), hsa-miR-142a-5p and hsa-miR-151a-3p (P < 0.001) as well as between hsa-miR-146a-5p and hsa-miR-151a-3p (P < 0.001), Table 1.

**Table 1:** Correlations among miRNAs.

		hsa-miR-23a-3p	hsa-miR-29c-3p	hsa-miR-30b-5p	hsa-miR-142a-5p	hsa-miR-146a-5p	hsa-miR-151a-3p
hsa-miR-23a-3p	r_S_ P n	1	0.788 <0.001 85	0.854 <0.001 87	0.367 <0.001 87	0.306 0.005 83	0.679 <0.001 87
hsa-miR-29c-3p	r_S_ P n	0.788 <0.001 85	1	0.723 <0.001 87	−0.012 0.915 87	−0.059 0.596 83	0.310 0.004 87
hsa-miR-30b-5p	r_S_ P n	0.854 <0.001 87	0.723 <0.001 87	1	0.218 0.040 89	0.203 0.062 85	0.542 <0.001 89
hsa-miR-142a-5p	r_S_ P n	0.367 <0.001 87	−0.012 0.915 87	0.218 0.040 89	1	0.774 <0.001 85	0.788 <0.001 89
hsa-miR-146a-5p	r_S_ P n	0.306 0.005 83	−0.059 0.596 83	0.203 0.062 85	0.774 <0.001 85	1	0.810 <0.001 85
hsa-miR-151a-3p	r_S_ P n	0.679 <0.001 87	0.310 0.0045 87	0.542 <0.001 89	0.788 <0.001 89	0.810 <0.001 85	1

*Note: The values represent the Spearman’s correlation coefficient (r*_*S*_ *), the p-value of its test of significance (P) and the number of patients (n).*

In comparison to the established biomarkers of dementia, statistically significant correlations were identified between the plasmatic Aβ 1–42 and four miRNAs: has-miR-29c-3p, hsa-miR-142a-5p, hsa-miR-146a-5p, and hsa-miR-151a-3p. Similar correlations were also identified in relation to the plasmatic Aβ1–42/Aβ1–40, Table 2.

### Analysis of dependence of selected biomarkers and diagnostic groups

**Fig 1:**
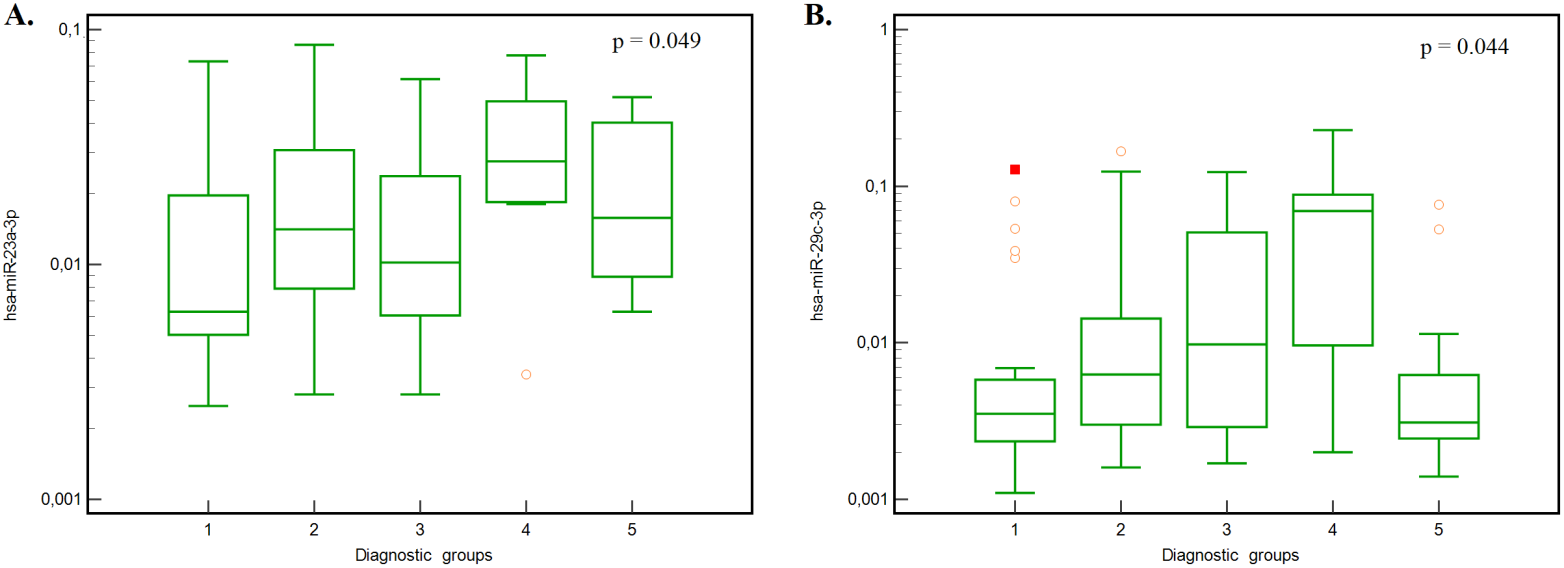
Concentration of hsa-miR-23a-3p and hsa-miR-29c-3p in different groups. According to the graphs, there appears to be a difference between group 3 (movement disorders without dementia) and group 4 (movement disorders with dementia). However, in both cases, the results were insignificant (for hsa-miR-23a-3p, P = 0.0752, for hsa-miR-29c-3p, P = 0.1823). See Tables 4 and 5.

The Kruskal-Wallis test revealed statistically significant differences in hsa-miR-23a-3p (P = 0.049) and hsa-miR-29c-3p (P = 0.043) among the different diagnostic groups (Table 3 and Fig 1). The post-hoc Conover test, utilised to ascertain specific between-group disparities, indicated statistical significance between group 1 (AD) and group 4 (combination of cognitive syndrome and movement disorders) and group 5 (healthy controls) for hsa-miR-23a-3p.

**Table 4:** Comparison of group 3 and 4 using hsa-miR-29c-3p and hsa-miR-23a-3p.

miRNA		Group 3	Group 4
	**Sample size**	18	8
**hsa-miR-29c-3p**	**Lowest value**	0.0017	0.0020
	**Highest value**	0.1232	0.2283
	**Median**	0.0110	0.0696
	**95% CI for the median**	0.0030 to 0.0468	0.0065 to 0.1272
	**Interquartile range**	0.0029 to 0.0507	0.0099 to 0.0895
**hsa-miR-23a-3p**	**Lowest value**	0.0028	0.0034
	**Highest value**	0.0616	0.0777
	**Median**	0.0102	0.0276
	**95% CI for the median**	0.0062 to 0.0235	0.0153 to 0.0709
	**Interquartile range**	0.0061 to 0.0238	0.0185 to 0.0524

*Note: Group 3: Parkinson’s Disease; 4: combination of dementia and movement disorder*.

**Table 5:** Mann-Whitney test (independent samples) of hsa-miR-29c-3p and hsa-miR-23a-3p in group 3 and 4.

	hsa-miR-29c-3p	hsa-miR-23a-3p
**Average rank of group 3**	12.1667	11.1765
**Average rank of group 4**	16.500	16.8750
**Mann-Whitney U**	48.00	37.00
**Two-tailed probability**	P = 0.1823	P = 0.0752

*Note: Group 3: Parkinson’s Disease; 4: combination of dementia and movement disorder*.

Statistical significance was found between group 1 and group 4, and between group 4, group 1 and group 5 and between group 5 and group 4 for hsa-miR-29c-3p.

### Diagnostic accuracy of studied biomarkers

Healthy controls exhibited significantly lower median serum NfL, CSF NfL, CSF Aβ 1–42, CSF p-tau, CSF t-tau levels in comparison to patients (p < 0.01, Mann-Whitney test), as outlined in S2 File.

**Fig 2:**
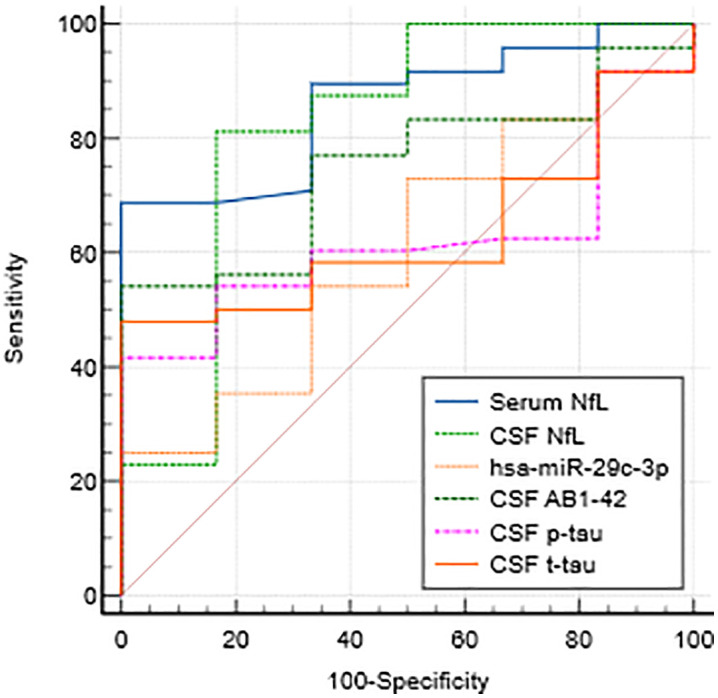
Analysis of diagnostic accuracy of selected biomarkers with the ROC curve.

Six biomarkers (serum NfL, CSF NfL, hsa-mir-29c-3p, CSF Aβ 1–42, CSF p-tau, CSF t-tau) demonstrated the capacity to predict neurodegenerative processes with adequate diagnostic efficacy (AUC > 0.8, Table 6, Fig 2). ROC analysis demonstrated that the optimal cut-off value for the selection of biomarkers for the identification of patients is 16.9 ng/L for serum NfL, with 73.1% sensitivity and 92.3% specificity, and 777 ng/L for CSF NfL, with 74.6% sensitivity and 75.0% specificity, 0.0038 for hsa-miR-29c-3p with 59.7% sensitivity and 69.2% specificity, and 937.5 ng/l for CSF Aβ 1–42 with 50.8% sensitivity and % sensitivity and 94.4% specificity, 57.2 ng/L for CSF p-tau with 43.7% sensitivity and 100% specificity, and 368 ng/L for CSF t-tau with 51.5% sensitivity and 94.4% specificity. Concurrently, the integration of conventional biomarkers (serum NfL, CSF NfL, CSF Aβ 1–42, CSF t-tau, and CSF p-tau) with hsa-miR-29c-3p enhanced the overall diagnostic efficacy, attaining an area under the curve (AUC) of 0.917.

**Table 6:** Evaluation of the diagnostic performance of selected biomarkers using ROC curve analysis.

Variable	AUC	SE ^a^	95% CI ^b^
Serum NfL	0.894	0.0665	0.695 to 0.983
CSF NfL	0.867	0.0833	0.661 to 0.971
hsa-miR-29c-3p	0.800	0.0927	0.582 to 0.936
CSF Aβ 1–42	0.716	0.0833	0.577 to 0.830
CSF p-tau	0.670	0.0762	0.529 to 0.792
CSF t-tau	0.668	0.0796	0.527 to 0.790

*Note:* ^*a*^ *Hanley & McNeil, 1982;* ^*b*^ *Binomial exact*.

## Discussion

Many studies have reported the dysregulation of various miRNAs in neurodegenerative diseases. Latest studies have identified plasma miRNAs that reflect A/T/N changes in people with dementia, and even miRNAs that can serve as predictive biomarkers for the transition from MCI to AD [35,36]. A significant decrease in the plasma extracellular vesicle-associated hsa-miR-23a-3p has been observed in AD patients when compared to healthy controls [23]. However, the study conducted by Serpente et al. found a significant upregulation in patients with AD [24]. In our study, we observed a decrease in the regulation of this miRNA in patients with AD. In contrast, an increased regulation was noted in individuals with both dementia and parkinsonism. Differences from previous studies may stem from microRNA origins; some focused on extracellular vesicle-associated miRNAs, while we analyzed total circulating miRNAs, which may reflect broader but less specific signals. These discrepancies could also be due to variations in disease stage at sampling, cohort composition, comorbidities, age factors, and differences in pre-analytical handling and normalization, leading to inconsistent results.

It has been suggested that the diminished expression of hsa-miR-23a-3p contributes to synaptic damage by disrupting the regulation of SNAP-25 [37]. Additionally, it activates pro-apoptotic proteins, including PUMA, Noxa, and Bax, while enhancing neuroinflammation through the PTEN/AKT/mTOR signaling pathway [38,39]. It is noteworthy that we found no correlation among the typical Alzheimer’s biomarkers, including Aβ, p-tau, and t-tau. Furthermore, this miRNA exhibited a correlation with hsa-miR-29c-3p, hsa-miR-30b-5p, and hsa-miR-151a-3p.

Our data indicates a correlation between levels of hsa-miR-29c-3p and plasmatic Aβ42, highlighting a trend that distinguishes AD from other groups. The literature suggests that this microRNA is downregulated in the brains of mice with AD. It is proposed that miR-29c-3p may play a role in Aβ neurotoxicity by affecting the activity of the beta-site amyloid precursor protein cleaving enzyme 1 (BACE1) [29]. Furthermore, miR-29c-3p participates in the modulation of neuroinflammation in AD via the PRR34-AS1/miR-29c-3p axis [40]. In groups 3 and 4, which are mainly classified as synucleinopathies, the concentration of miR-29c-3p has been found to be elevated. Several studies have reported deregulation of this microRNA in the brains of individuals with Parkinson’s disease (PD), multiple system atrophy (MSA), as well as in the blood of those with isolated rapid eye movement sleep behavior disorder (IRBD) and Lewy body dementia (LBD). However, these findings indicate an opposing trend [31,32,41]. The explanation for our findings may be related to the diverse stages of disease present in our patients. The referenced articles primarily focused on early disease stages, while our patients had been under observation for an extended period during which we tested their blood. Although we found no correlation between hsa-miR-29c-3p and α-synuclein in cerebrospinal fluid (CSF), it is known that this biomarker lacks sensitivity in detecting synucleinopathies when examined in bodily fluids. The only significant correlation observed was between miR-29c-3p and the Aβ42/Aβ40 ratio.

We also established a significant correlation between hsa-miR-30b-5p and hsa-miR-29c-3p, as well as between hsa-miR-23a-3p and hsa-miR-151a-3p, but no dependency was found with other biomarkers. Similar expression patterns were observed between hsa-miR-142a-5p and hsa-miR-146a-5p, which also strongly correlated with Aβ42 and the Aβ42/Aβ40 ratio. Sierksma et al. mentioned both of these microRNAs in their discussion of the pathological processes in AD [25]. In hsa-miR-151a-3p, a correlation is observed with every studied miRNA except hsa-miR-29c-3p, and there is no correlation with other biomarkers.

The results indicate that hsa-miR-29c-3p is a promising miRNA; however, compared to other biomarkers, it did not enhance the diagnostic accuracy of more conventional neurodegeneration biomarkers. To differentiate between patient groups, we examined hsa-miR-29c-3p and hsa-miR-23a-3p as a potential prognostic biomarker for cognitive decline in movement disorders, comparing groups 3 and 4. Unfortunately, the results did not reach statistical significance, which may be attributed to the unequal number of patients in each group.

Higher levels of miR-23a-3p may inhibit inflammation and offer neuroprotection [39,42]. Likewise, increased miR-29c-3p levels can reduce TET2 expression and lessen autophagy [32]. These molecules hold promise as therapeutic agents.

Recent research highlights miRNAs as diagnostic, prognostic, and therapeutic response modulators. They are increasingly discussed as mediators of personalized nutrition approaches, where individual dietary interventions can influence disease-related molecular pathways through miRNA regulation. Using intravenous trehalose and even nutritional interventions like ketogenic diet influence miRNA profiles linked to synaptic plasticity and neuroinflammation [43–45].

Our study has several limitations. Firstly, we encountered a shortage of available samples for analysis within the patient groups and among the healthy controls. Additionally, the composition of the patient groups was not entirely uniform; each group included individuals at various stages of the disease, and Groups 2 and 4 encompassed several distinct nosological entities. Larger samples would enhance the validity.

Another limitation is the nature of miRNAs themselves. These biomarkers are being studied in various medical fields, and their regulation can be affected by illnesses or conditions beyond just neurodegenerative diseases. Additionally, physical stimuli can epigenetically influence miRNA regulation; for example, listening to music or exposure to radiation may have an impact [26,46]. Therefore, the age differences among our group members may also influence how they are affected by the long-term exposure to the factors mentioned above. Perhaps we could collect additional information about our subjects’ lifestyles and comorbidities to obtain more accurate results. However, this might lead to more inconclusive findings.

A common limitation in research on neurodegenerative diseases is the uncertainty associated with diagnoses. Although AD has recognizable biomarker patterns that enable more reliable diagnoses, definitive diagnoses for other neurodegenerative diseases are usually only possible after death. Furthermore, autopsy findings often reveal that multiple pathologies are present simultaneously [47]. Additionally, although the roles of inflammatory markers and age were examined, the limited systematic data on comorbidities and the weak correlations limited the ability to fully adjust for potential confounders.

## Conclusion

The microRNAs that exhibit the greatest potential for differentiating between neurodegenerative diseases are hsa-miR-23a-3p and hsa-miR-29c-3p. Moreover, hsa-miR-29c-3p has been observed to correlate with Aβ42 levels and the Aβ42/Aβ40 ratio. Although additional comprehensive research is required, there exists a possibility for the utilization of this microRNA as a future therapeutic agent.

## Supporting information

S1 Fig A visual diagram of the selection process of microRNA targets identification. (TIF)

S2 Fig A simple workflow diagram of the methodology for the assay of selected miRNAs and other biomarkers. (TIF)

S1 File Results of identification of potential microRNA targets. (PDF)

S2 File Data of patients. (XLSX)
