# Supplementary figures and images for: Identification and evaluation of potential microRNA markers for diagnostics in neurodegenerative diseases and correlation with other biochemical markers
Source: PLoS One. 2025 Oct 10;20(10):e0333801. doi: 10.1371/journal.pone.0333801 (PMC12513651; doi:10.1371/journal.pone.0333801)

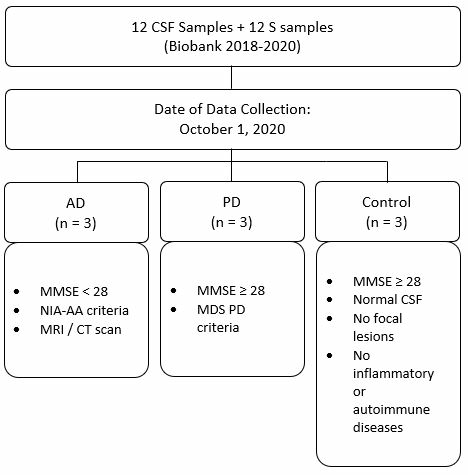

Supplement: S1 Fig — (TIF) [file pone.0333801.s001.tif]

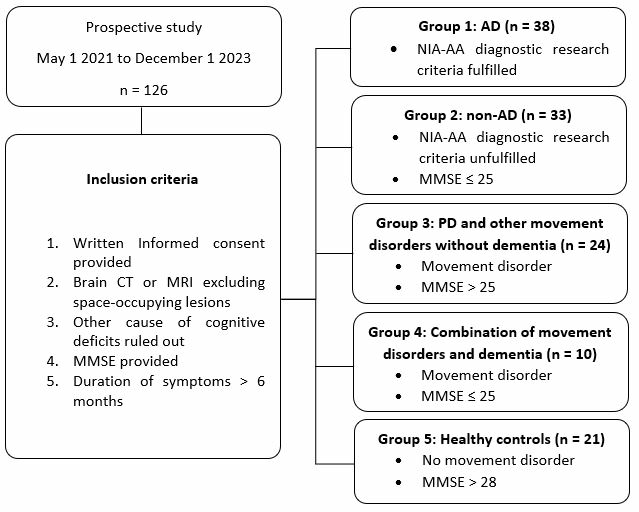

Supplement: S2 Fig — (TIF) [file pone.0333801.s002.tif]
