# Supplementary material for: Identification and evaluation of potential microRNA markers for diagnostics in neurodegenerative diseases and correlation with other biochemical markers
Source: PLoS One. 2025 Oct 10;20(10):e0333801. doi: 10.1371/journal.pone.0333801 (PMC12513651; doi:10.1371/journal.pone.0333801)
Supplement: S1 File — (PDF) [file pone.0333801.s003.pdf]

miRNA screening: serum

Dementia vs. controls

Table 1. Selection of results for dementia serum vs. controls

miRNA	Dementia diameter	Average Checks	mean difference	fold-change final (Dementia vs. Controls)	p-value
hsa-miR-497-5p	1,52	2,47	-0,96	**1,94**	0,05
hsa-miR-328-3p	2,48	3,18	-0,70	**1,62**	**0,03***
hsa-miR-4732-3p	3,35	3,98	-0,63	**1,55**	0,05
hsa-miR-185-5p	-1,48	-2,07	0,59	**-1,50**	0,05
hsa-miR-142-5p	-2,14	-2,81	0,66	**-1,58**	**0,03***
hsa-miR-195-5p	4,41	3,61	0,79	**-1,73**	**0,03***
hsa-miR-143-3p	-0,15	-0,97	0,82	**-1,76**	0,05
hsa-miR-196b-5p	5,34	4,40	0,93	**-1,91**	**0,03***
hsa-miR-140-3p	2,74	1,61	1,13	**-2,19**	0,05
hsa-miR-144-3p	-2,99	-4,31	1,32	**-2,50**	0,05
hsa-miR-151a-3p	-0,75	-0,23	-0,52	**1,43**	**0,03***
hsa-miR-199a-3p	-2,25	-1,82	-0,43	**1,34**	**0,03***
hsa-miR-423-3p	-0,15	0,25	-0,40	**1,32**	**0,03***
hsa-miR-30b-5p	-0,60	-0,37	-0,23	**1,17**	**0,03***
hsa-miR-324-5p	2,53	2,11	0,42	**-1,34**	0,05
hsa-let-7i-5p	-0,87	-1,33	0,46	**-1,38**	0,05
hsa-miR-324-3p	1,69	1,20	0,49	**-1,41**	0,05
hsa-miR-29c-3p	-2,65	-3,19	0,53	**-1,45**	**0,03***

up-regulation in patients with dementia (more than 1.5 times)

down-regulation in patients with dementia (more than 1.5 times)

*** p-value <0.05**

Parkinson vs. Controls

Table 2. Selection of results for Parkinson's serum vs. controls

miRNA	Parkinson diameter	Average Checks	mean difference	fold-change final (Parkinson vs. Controls)	p-value
hsa-miR-4732-3p	3,01	3,98	-0,97	**1,96**	**0,03***
hsa-miR-222-3p	-5,89	-5,05	-0,84	**1,79**	**0,03***
hsa-miR-133a-3p	-3,23	-2,49	-0,74	**1,67**	0,05
hsa-miR-625-5p	-3,23	-2,53	-0,69	**1,62**	**0,03***
hsa-miR-199a-5p	-2,56	-1,89	-0,68	**1,60**	**0,03***
hsa-miR-155-5p	2,27	2,84	-0,56	**1,48**	**0,03***
hsa-miR-320c	-2,02	-1,49	-0,53	**1,44**	**0,03***
hsa-miR-142-5p	-1,85	-2,81	0,95	**-1,94**	**0,03***
hsa-miR-224-5p	-8,21	-7,69	-0,52	**1,44**	0,05
hsa-miR-127-3p	3,99	4,50	-0,51	**1,43**	0,05
hsa-miR-340-5p	0,37	0,00	0,37	**-1,29**	**0,03***

up-regulation in patients with Pakinson's (more than 1.5 times)
down-regulation in Parkinson's patients (more than 1.5 times)
*** p-value <0.05**

Patients (dementia+ Parkinson's) vs. Controls

Table 3. Selection of outcomes for serum patients vs. controls

miRNA	diameter Patients	diameter Controls	mean difference	fold-change final (patients vs. controls)	p-value
hsa-miR-4732-3p	3,18	3,98	-0,80	**1,75**	**0,01***
hsa-miR-497-5p	1,74	2,47	-0,74	**1,67**	**0,03***
hsa-miR-122-5p	-2,65	-1,94	-0,71	**1,64**	p>0,05
hsa-miR-616-3p	5,20	5,91	-0,70	**1,63**	p>0,05
hsa-miR-199a-5p	-2,54	-1,89	-0,65	**1,57**	**0,02***
hsa-miR-19a-3p	-4,87	-4,23	-0,64	**1,56**	p>0,05
hsa-miR-139-3p	3,64	4,28	-0,64	**1,56**	p>0,05
hsa-miR-222-3p	-5,68	-5,05	-0,63	**1,55**	0,05
hsa-miR-625-5p	-3,13	-2,53	-0,60	**1,51**	0,05
hsa-miR-99a-5p	0,86	1,45	-0,59	**1,51**	p>0,05
hsa-miR-28-3p	-3,03	-2,46	-0,58	**1,49**	0,05
hsa-miR-181a-5p	-1,83	-1,35	-0,49	**1,40**	0,05
hsa-miR-224-5p	-8,16	-7,69	-0,48	**1,39**	0,05
hsa-miR-151a-3p	-0,61	-0,23	-0,38	**1,30**	0,05
hsa-miR-152-3p	2,24	2,60	-0,36	**1,28**	0,05
hsa-miR-30b-5p	-0,59	-0,37	-0,22	**1,16**	0,05
hsa-miR-340-5p	0,32	0,00	0,31	**-1,24**	0,05
hsa-miR-29c-3p	-2,72	-3,19	0,47	**-1,39**	0,05
hsa-miR-195-5p	4,20	3,61	0,58	**-1,50**	**0,01***
hsa-miR-185-5p	-1,46	-2,07	0,62	**-1,54**	0,05
hsa-miR-196b-5p	5,11	4,40	0,70	**-1,63**	**0,02***
hsa-miR-142-5p	-2,00	-2,81	0,81	**-1,75**	**0,01***

up-regulation in patients (more than 1.5 times)

down-regulation in patients (more than 1.5 times)

*** p-value <0.05**

Dementia vs. Parkinson

Table 4. Selection of results for dementia vs. Parkinson's serum

miRNA	diameter Dementia	diameter parkinson	mean difference	fold-change	fold-change final (Demece vs. Parkinson)	p-value
hsa-miR-409-3p	1,18	2,50	-1,32	2,50	**2,50**	0,19
hsa-miR-495-3p	1,81	2,90	-1,10	2,14	**2,14**	0,66
hsa-miR-374b-5p	-1,04	-0,02	-1,01	2,02	**2,02**	0,08
hsa-miR-330-3p	4,24	5,18	-0,94	1,92	**1,92**	0,19
hsa-let-7f-5p	1,85	2,77	-0,92	1,89	**1,89**	0,19
hsa-miR-339-5p	2,17	3,07	-0,90	1,87	**1,87**	0,08
hsa-miR-487b-3p	2,82	3,72	-0,90	1,86	**1,86**	0,66
hsa-miR-28-5p	2,38	3,16	-0,78	1,72	**1,72**	0,08
hsa-miR-30c-5p	-0,69	0,05	-0,74	1,67	**1,67**	0,08
hsa-miR-122-5p	-3,02	-2,28	-0,74	1,67	**1,67**	0,66
hsa-miR-191-5p	-2,41	-1,67	-0,74	1,67	**1,67**	0,19
hsa-miR-199b-3p	-2,46	-1,73	-0,72	1,65	**1,65**	0,38
hsa-miR-337-5p	2,87	3,56	-0,69	1,61	**1,61**	0,66
hsa-miR-32-5p	3,54	4,22	-0,68	1,60	**1,60**	0,38
hsa-miR-328-3p	2,48	3,15	-0,67	1,59	**1,59**	0,66
hsa-miR-199a-3p	-2,25	-1,61	-0,64	1,56	**1,56**	0,08
hsa-miR-326	1,52	2,12	-0,60	1,52	**1,52**	0,08
hsa-miR-21-3p	4,08	4,67	-0,59	1,50	**1,50**	0,08
hsa-miR-140-3p	2,74	2,11	0,63	0,65	**-1,54**	0,38
hsa-miR-1285-5p	0,88	0,23	0,64	0,64	**-1,56**	0,66
hsa-miR-424-5p	-0,52	-1,18	0,66	0,63	**-1,58**	0,38
hsa-miR-15b-3p	1,77	1,10	0,68	0,63	**-1,60**	0,38
hsa-miR-451a	-9,67	-10,36	0,69	0,62	**-1,62**	0,66
hsa-miR-133a-3p	-2,51	-3,23	0,72	0,61	**-1,65**	0,38
hsa-miR-616-3p	5,59	4,82	0,76	0,59	**-1,70**	0,19
hsa-miR-1290	-6,95	-7,71	0,76	0,59	**-1,70**	0,38
hsa-miR-627-5p	4,35	3,56	0,79	0,58	**-1,73**	0,19
hsa-miR-92a-3p	-3,10	-3,95	0,85	0,56	**-1,80**	0,38
hsa-miR-1224-3p	5,00	4,10	0,90	0,54	**-1,86**	0,19
hsa-miR-1285-3p	5,96	4,68	1,27	0,41	**-2,42**	0,19

up-regulation in patients with dementia (more than 1.5 times)

down-regulation in patients with dementia (more than 1.5 times)

*** p-value <0.05**

Endogenous controls - serum

GeNorm and Normfinder were used to select endogenous controls from the measured data.

GeNorm - histogram

The following graphical representation expresses the frequency of M-value (M-value AntiLog2 - Cq values were used for the analysis) for each microRNA. The miRNAs with the lowest M-value - the most suitable endogenous controls - are shown in red.

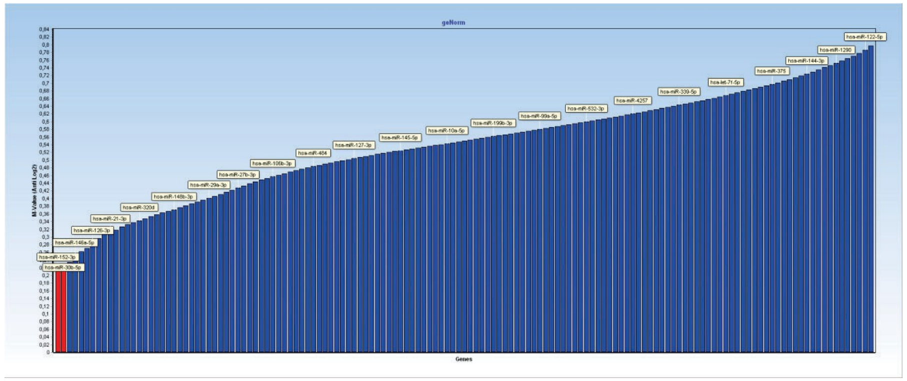

Normfinder - histogram

The following graphical representation expresses the SD frequency for each microRNA. The miRNAs with the lowest SD - the most suitable endogenous controls - are shown in red.

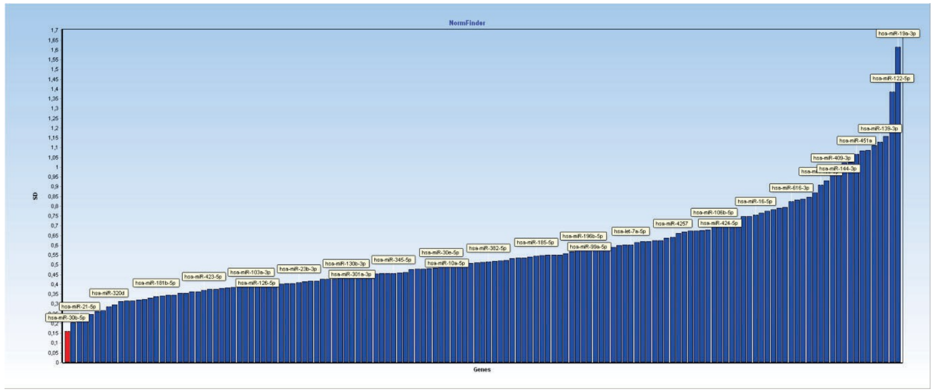

Table 5. Selection of the most suitable endogenous miRNAs for normalization of serum samples

Gene Name	order GeNorm	order NormF	sum of o r d e r
hsa-miR-30b-5p	2	1	3
hsa-miR-26a-5p	3	4	7
hsa-miR-22-5p	6	2	8
hsa-miR-152-3p	1	9	10
hsa-miR-146b-5p	5	5	10
hsa-miR-146a-5p	4	7	11
hsa-miR-423-3p	8	11	19
hsa-miR-126-3p	7	15	22
hsa-miR-320d	15	8	23
hsa-miR-374a-5p	13	12	25

Conclusion: a total of 141 miRNAs were analyzed for the selection of endogenous control for the serum matrix Targets.

Serial numbers from GeNorm and Normfinder were summed, with 10 listed in the table miRNA targets with the lowest rank sum - potentially the most suitable endogenous controls.

For further selection for the validation study, it is recommended to take into account in particular the concentration of the miRNAs in the studied matrix.

CSF miRNA screening

Dementia vs. controls

Table 6: Selection of outcomes for CSF dementia vs. controls

miRNA	diameter DEMENCE	diameter CONTROLS	mean difference	fold-change final (demece vs. Controls)	p-value
hsa-miR-125b-5p	1,09	2,29	-1,19	**2,29**	0,70
hsa-miR-320c	1,96	2,57	-0,61	**1,52**	0,37
hsa-miR-122-5p	4,46	3,87	0,59	**-1,50**	**0,05***
hsa-miR-409-3p	3,09	2,31	0,78	**-1,71**	0,25

up-regulation in patients with dementia (more than 1.5 times)

down-regulation in patients with dementia (more than 1.5 times)

*** p-value= 0.05**

Parkinson vs. Controls

Table 8: Selection of outcomes for CSF Parkinson vs. controls

miRNA	diameter PARKINSON	diameter Controls	mean difference	fold-change final (Parkinson vs. Controls)	p-value
hsa-miR-148b-3p	2,30	3,09	-0,79	**1,73**	0,16

up-regulation in Parkinson's patients (more than 1.5 times)

CONCLUSION: There was no significant difference between the Parkinson's and control groups. The table shows miR-148-3p, which shows a difference of more than 1.5-fold, but with very low confidence in the result.

3.4.3 Patients (dementia+ Parkinson's) vs. controls

Table 9: Selected outcomes for CSF patients (dementia+parkinson's) vs. controls

miRNA	diameter PATIENTS	diameter CONTROLS	mean difference	fold-change final (demece vs. Controls)	p-value
hsa-miR-125b-5p	1,66	2,29	-0,63	**1,55**	p> 0,05
hsa-miR-224-5p	-8,85	-8,68	-0,17	**1,12**	**0,045***
hsa-miR-122-5p	4,28	3,87	0,40	**-1,32**	**0,045***

up-regulation in patients (more than 1.5 times)
down-regulation in patients (more than 1.5 times)
*** p-value <0.05**

Conclusion: miRNA targets recommended for further selection for the validation study are listed in the table. The best result is the most significant fold-change and the lowest p-value.

3.4.4 Dementia vs. Parkinson

Table 10: Selection of results for CSF Dementia vs. Parkinson

miRNA	PARKINSON diameter	DEMENCE diameter	mean difference	fold-change final (Parkinson vs. Dementia)	p-value
hsa-miR-409-3p	1,78	3,09	-1,31	**2,47**	p>0,05
hsa-miR-487b-3p	3,62	4,67	-1,05	**2,07**	p>0,05
hsa-miR-148b-3p	2,30	3,23	-0,94	**1,91**	p>0,05
hsa-miR-425-3p	3,74	4,32	-0,59	**1,50**	p>0,05
hsa-miR-320c	3,04	1,96	1,08	**-2,11**	p>0,05
hsa-miR-125b-5p	2,22	1,09	1,12	**-2,18**	p>0,05

up-regulation in Parkinson's patients (more than 1.5 times)
down-regulation in Parkinson's patients (more than 1.5 times)
*** p-value <0.05**

Conclusion: miRNA targets recommended for further selection for the validation study are listed in the table. The best result is the most significant fold-change and the lowest p-value. The results do not contain any statistically significant results, mainly due to the comparison of very few samples (3+3).

3.5 Endogenous controls - CSF

GeNorm and Normfinder were used to select endogenous controls from the measured data.

GeNorm - histogram

The following graphical representation expresses the frequency of M-value (M-value AntiLog2 - Cq values were used for the analysis) for each microRNA. The miRNAs with the lowest M-value - the most suitable endogenous controls - are shown in red.

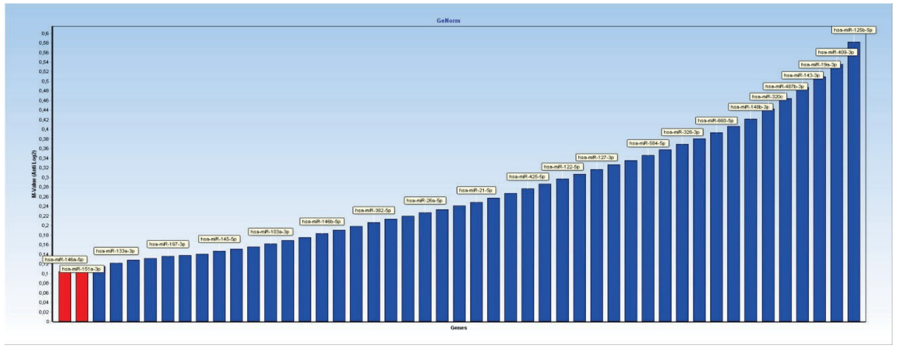

Normfinder - histogram

The following graphical representation expresses the SD frequency for each microRNA. The miRNAs with the lowest SD - the most suitable endogenous controls - are shown in red.

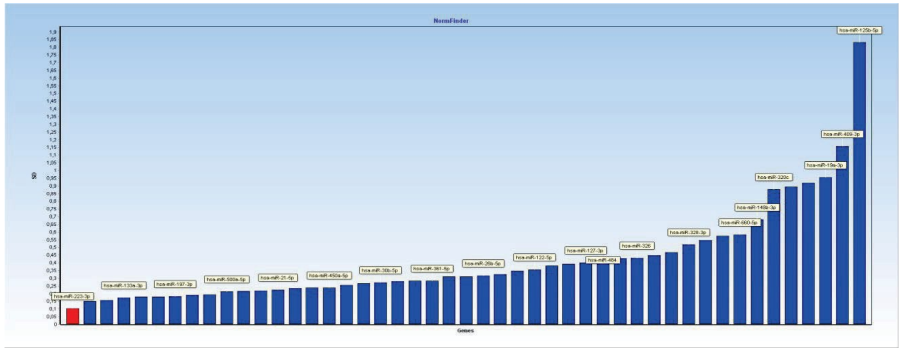

Table 11: Selection of the most suitable endogenous miRNAs for normalization of CSF samples

Gene Name	GeNorm ranking	NormF ranking	sum of rank
hsa-miR-151a-3p	2	2	4
hsa-miR-223-3p	3	2	5
hsa-miR-133a-3p	4	4	8
hsa-miR-146a-5p	1	8	9
hsa-miR-625-5p	5	6	11
hsa-miR-224-5p	9	3	12
hsa-miR-197-3p	7	7	14
hsa-miR-28-3p	6	9	15
hsa-miR-500a-5p	8	10	18
hsa-miR-15b-5p	15	5	20

Z
